# Risk of Rebleeding and Mortality in Cirrhotic Patients with Peptic Ulcer Bleeding: A 12-Year Nationwide Cohort Study

**DOI:** 10.1371/journal.pone.0168918

**Published:** 2017-01-12

**Authors:** Shih-Cheng Yang, Chien-Ning Hsu, Chih-Ming Liang, Wei-Chen Tai, Cheng-Kun Wu, Chih-Wei Shih, Ming-Kun Ku, Lan-Ting Yuan, Jiunn-Wei Wang, Kuo-Lun Tseng, Tsung-Hsing Hung, Seng-Howe Nguang, Pin-I Hsu, Deng-Chyang Wu, Seng-Kee Chuah

**Affiliations:** 1 Division of Hepatogastroenterology, Department of Internal Medicine, Kaohsiung Chang Gung Memorial Hospital, Kaohsiung, Taiwan; 2 Department of Pharmacy, Kaohsiung Gang Gung Memorial Hospital, Kaohsiung, Taiwan; School of Pharmacy, Kaohsiung Medical University, Kaohsiung, Taiwan; 3 Chang Gung University, College of Medicine, Kaohsiung, Taiwan; 4 Division of Hepatogastroenterology, Department of Internal Medicine, Chia-Yi Chang Gung Memorial Hospital, Chia-Yi, Taiwan; 5 Division of Gastroenterology, Fu-Ying University Hospital, Pin-Tung, Taiwan; 6 Division of Gastroenterology, Yuan General Hospital, Kaohsiung, Taiwan; 7 Division of Gastroenterology, Department of Internal Medicine, Kaohsiung Medical University Hospital and Kaohsiung Medical University, Kaohsiung, Taiwan; 8 Division of Hepatogastroenterology, Department of Internal Medicine, Buddhist Tzu Chi General Hospital, Dalin Branch, Chia-Yi, Taiwan; 9 Division of Gastroenterology, Pin-Tung Christian Hospital, Pin-Tung, Taiwan; 10 Division of Gastroenterology, Department of Internal Medicine, Kaohsiung Veterans General Hospital, National Yang-Ming University, Kaohsiung, Taiwan; Cliniques Universitaires Saint-Luc, BELGIUM

## Abstract

Although a few studies have investigated the risks of peptic ulcer bleeding (PUB) in cirrhotic patients, large population-based studies on in-hospital and long-term reports on recurrent PUB in a cohort of cirrhotic patients are lacking. This 12-year nationwide cohort study aimed to investigate the risks of in-hospital and long-term rebleeding and mortality in cirrhotic patients and to identify possible risk factors. Patient data from 1997 to 2008 were extracted from the National Health Insurance Research Database in Taiwan. A total of 15,575 patients who were discharged with a diagnosis of PUB were identified after strict exclusions (n = 2889). Among them, patients with cirrhosis (n = 737) and those with chronic hepatitis (n = 1044) were compared to propensity-score matched normal controls at a ratio of 1:1. Accumulated in-hospital and long-term follow-up PUB-free survival rates were analyzed in patients with cirrhosis, patients with chronic hepatitis, and matched controls. Cox proportional hazards regression was used to identify each independent risk factor. Compared with matched controls, patients with cirrhosis exhibited a 2.62-fold (95% CI: 1.74–3.92) higher risk of developing in-hospital rebleeding, but the risk of long-term rebleeding was comparable between cirrhotic patients and matched controls (hazard ratio: 1.29, 95% CI: 0.8–2.09). On the other hand, no significant difference was observed in in-hospital and long-term rebleeding between chronic hepatitis patients and matched controls. We compared the survival rates of cirrhotic and chronic hepatitis patients to that of matched controls. After propensity score matching, both cirrhotic and chronic hepatitis patients showed significantly lower survival than the matched controls (P < 0.0001 and 0.033, respectively) during the 12-year follow-up period. However, in-hospital and long-term rebleeding rates were not significantly different between chronic hepatitis patients and matched controls (P = 0.251 and 0.474, respectively). In conclusion, liver cirrhosis increased health care expenses in patients with PUB and these patients exhibited higher recurrent bleeding rate than non-cirrhotic patients during hospitalization. Cirrhosis and chronic hepatitis are independently associated with an increased long-term mortality when compared with patients without liver disease.

## Introduction

Peptic ulcer bleeding (PUB) is an important cause of hospitalization worldwide [1, 2]. Advanced liver diseases may result in coagulation defects and susceptibility to hemodynamic disturbances and infections. Occurrence of peptic ulcers in patients with liver cirrhosis can become a serious problem. Cirrhotic patients are at risk for both variceal and non-variceal causes of upper gastrointestinal (UGI) bleeding. PUB accounts for 30%–40% of nonvariceal UGI bleeding in cirrhotic patients [3]. Cirrhotic patients in clinical practice are associated with more frequent PUB and have higher mortality rates than those without cirrhosis when they bleed [4–7]. In addition, 15% of patients with cirrhosis die within 6 weeks after nonvariceal UGI bleeding [7]. To date, large population-based studies on in-hospital and long-term reports on recurrent PUB and mortality in a cohort of cirrhotic patients are lacking. This 12-year nationwide cohort study aimed to investigate the risks of in-hospital and long-term rebleeding and mortality in cirrhotic patients and to identify possible risk factors.

## Materials and Methods

### Ethics statement

The study protocol was approved by the institutional review board and the Ethics Committee of Chang Gung Memorial Hospital (IRB104-9779B). The Ethics Committee waived the requirement for informed consent for this study, and all the data were analyzed anonymously.

### Data source

The National Health Insurance (NHI) program in Taiwan was established in 1995 and covers 99% of Taiwan’s population of 23 million. In the present study, 1,000,000 individuals, approximately 5% of Taiwan’s population, were randomly selected from the 2000 Registry for Beneficiaries of the National Health Insurance Research Database (NHIRD) [8]. The cohort data of individuals from 1997 to 2009 included enrolment files, claims data, appalling illness files, and drug prescription registry. In the cohort dataset, each patient’s original identification number was anonymized and de-identified prior to retrieval of data for privacy purposes. The International Classification of Diseases, Ninth Revision, Clinical Modification (ICD-9-CM) was used to define diseases. The data analysts were staff of Kaohsiung Medical Center, a site of the Collaboration Center of Health Information Application, Ministry of Health and Welfare

### Study groups and inclusion and exclusion criteria

**Fig 1:**
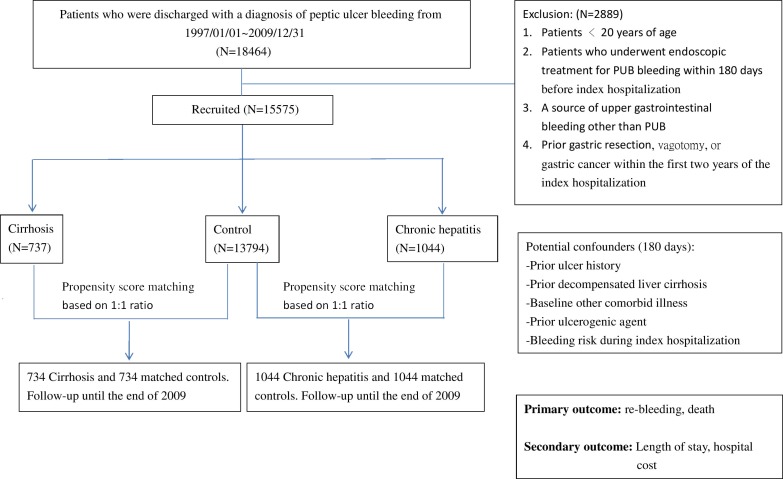
Schematic flowchart of study design.

All discharged patients aged 20 years or older at admission and with primary diagnosis code at discharge according to ICD-9-ICM of PUB (codes: 531.0, 531.2, 531.4, 531.6, 532.0, 532.2, 532.4, 532.6, 533.0, 533.2, 533.4, and 533.6) were included. Fig 1 shows the schematic flowchart of study design. Patients with PUB (n = 18464) were obtained from the dataset. Two thousand eight-hundred and eighty-nine patients were excluded because of the following reasons: <20 years of age, coexisting source of UGI bleeding other than PUB as the primary hospitalization claims, prior history of gastric resection or vagotomy, received endoscopic treatment for PUB within 180 days before index hospitalization, and gastric cancer within one year prior to the index hospitalization. A total of 15,575 hospitalized patients with PUB were included in the final analysis. These patients were then divided into three groups. The first group consisted of patients with liver cirrhosis (ICD-9-CM codes: 571.2, 571.5, and 571.6) diagnosed in previous hospitalization claims or at least two successive claims at out-patient clinics before the index PUB hospitalization (classified as cirrhosis group, N = 737). The second group comprised patients with chronic hepatitis (ICD-9-CM codes: 571.4, 571.40, 571.49, 571.8, and 571.9) but without cirrhosis before and after enrolment (chronic hepatitis group, N = 1044). The third group consisted of patients who were discharged with a primary diagnosis of PUB with neither cirrhosis nor chronic hepatitis before and after enrolment (control group, N = 13794). Demographic information was obtained from the dataset, including all the comorbid illnesses recorded in the diagnoses for hospitalization. Patients with decompensated cirrhosis were those with a primary diagnosis of PUB concomitant to the diagnosis of conditions such as hepatorenal syndrome (ICD-9 code: 572.4), ascites (ICD-9 code: 789.5), variceal hemorrhage(ICD-9 code: 456.0, 456.20), hepatic encephalopathy (ICD-9 code: 572.2), and jaundice (ICD-9 code: 782.4) [9, 10]. Patients without any of the listed secondary diagnoses were considered as compensated cirrhosis.

### Comorbidities, medications, and other covariates

The Deyo adaptation of the Charlson comorbidity index (CCI) was used to identify the load of illnesses with scores from 0 to 17[11]. Higher score indicates greater load in patients. The CCI with 17 comorbid conditions is a well-validated index of comorbidity adjusted for disease load [11]. In addition to the comorbidity scores of CCI, other factors that could affect the severity of bleeding, such as the presence of coagulopathy, were also evaluated. Primary diagnosis of PUB was made in all hospitalized patients who had undergone a panendoscopy during the inpatient stay. Indicators of severity of bleeding during the index hospitalization were the need of endoscopic intervention for initial bleeding episode, presence of shock or respiratory failure, and episodes of supportive interventions. For example, by using the billing code of Taiwan NHI program, the use of mechanical ventilation served as a proxy indicator for hemodynamic status and severity of illness as covariates in multivariate analysis of the outcomes measure. To preclude pre-existing peptic ulcer diseases from affecting the risk estimation, we defined prior ulcer history as a patient with a diagnosis of peptic ulcer within 180 days before the index date. Use of medications for *Helicobacter pylori* infection was identified within 180 days before the index date. Infection was defined as a patient who underwent antibiotic treatment during index hospitalization. The antisecretory drugs prescribed during the index hospitalization and ulcerogenic drugs within 90 days prior to the index hospitalization were also analyzed. The NHIRD contains prescription details, including doses, frequencies, dates, and administration routes.

### Outcomes

The primary outcomes were recurrent PUB and all-cause mortality during hospitalization and 12 years after the index hospitalization discharge. Within the index hospitalization, any record of a repeated endoscopic intervention (billing code: 47043B), transcatheter arterial embolization (billing code: 33075B), and need for surgery due to bleeding (billing codes: 72006B, 72007B, 72009B, 72010B, 72011B, 72012B, 72018B, and 72019B) were defined as rebleeding events. The secondary outcomes were the length of stay, total hospital costs, and frequency of surgical intervention, transarterial embolization, and repeated endoscopic intervention in recurrent PUB.

### Statistical analysis

Continuous and categorical data are presented as mean ± standard deviation and as actual frequencies and percentages, respectively. Pearson’s chi-square or Fisher’s exact two-tailed test was performed to examine categorical data, and 2-sample t tests were conducted to assess continuous data between groups. Multivariate logistic regression was used to examine factors associated with primary outcome during the index hospitalization. Kaplan–Meier analysis and Cox proportional hazard regression were performed to compare the outcomes of interest between groups following the index hospital discharge. To minimize potential selection bias between groups, we employed propensity score matching (PSM) to establish comparison analyses. Covariates used to estimate the propensity score included age, gender, baseline individual disease conditions involved in the CCI algorithm, prior use of ulcerogenic drugs (aspirin, non-steroid anti-inflammatory drugs (NSAIDs), steroids, clopidogrel, ticlopidine, warfarin), prior ulcer history, and prior *Helicobacter pylori*(HP) therapy. The propensity score derived from the control group was matched with patients in the cirrhosis group at a 1:1 ratio (the same as matched with the chronic hepatitis group) using the greedy matching algorithm within the SAS software package [12]. All P-values were two-tailed, and values < 0.05 were considered statistically significant. All statistical analyses were conducted using the statistical software **S**AS (version 9.3; SAS Institute Inc., Cary, NC, USA).

## Results

Baseline demographics of patients with cirrhosis or chronic hepatitis were compared with those of the matched controls (Table 1). After matching by using the propensity score, patients with and those without liver diseases did not differ significantly in regard to any of the baseline characteristics in both comparison groups. With respect to severity of bleeding during hospitalization, although rare, patients with cirrhosis had a higher rate of coagulation defects than patients in the control group (1.09% versus 0.14%, P = 0.019). A low rate of coagulation defects revealed between chronic hepatitis group and its matched control group (0.57% versus 0%).

**Table 1:** Baseline characteristics of the study cohorts (propensity score-matched cohort).

Characteristics	Cirrhosis (n = 734)		Matched controls (n = 734)		P-value	Chronic hepatitis (n = 1044)		Control (n = 1044)		P-value
Age, years	60.98±14.63		62.05±16.04		0.184	59.35±16.16		60.86±17.30		0.039
Gender					0.863					0.962
Male	519	70.71%	522	71.12%		732	70.11%	733	70.21%	
Female	215	29.29%	212	28.88%		312	29.89%	313	29.98%	
Comorbid conditions*											
Acute myocardial infarction	6	0.82%	3	0.41%	0.316	15	1.44%	10	0.96%	0.314
Congestive heart failure	57	7.77%	55	7.49%	0.844	68	6.51%	83	7.95%	0.205
Peripheral vascular disease	15	2.04%	12	1.63%	0.560	7	0.67%	6	0.57%	0.781
Cerebral vascular accidents	72	9.81%	82	11.17%	0.394	146	13.98%	146	13.98%	1.000
Dementia	21	2.86%	19	2.59%	0.749	26	2.49%	27	2.59%	0.889
Pulmonary disease	145	19.75%	166	22.62%	0.180	210	20.11%	222	21.26%	0.517
Connective tissue disorder	10	1.36%	8	1.09%	0.635	24	2.30%	19	1.82%	0.441
Peptic ulcer	323	44.01%	356	48.50%	0.084	472	45.21%	495	47.41%	0.313
Diabetes	167	22.75%	177	24.11%	0.538	220	21.07%	215	20.59%	0.788
Diabetes complications	65	8.86%	62	8.45%	0.781	52	4.98%	48	4.60%	0.682
Paraplegia	12	1.63%	16	2.18%	0.445	16	1.53%	11	1.05%	0.333
Renal disease	89	12.13%	89	12.13%	1.000	102	9.77%	97	9.29%	0.709
Cancer	210	28.61%	204	27.79%	0.728	106	10.15%	97	9.29%	0.506
Metastatic cancer	30	4.09%	35	4.77%	0.526	19	1.82%	14	1.34%	0.314
Prior ulcer history (≤180 days)	80	10.90%	81	11.04%	0.933	62	5.94%	54	5.17%	0.445
Prior HP therapy (≤180 days)	8	1.09%	5	0.68%	0.403	4	0.38%	3	0.29%	0.705
**Prior use of ulcerogenic drugs (≤90 days)**										
Aspirin	70	9.54%	67	9.13%	0.788	115	11.02%	113	10.82%	0.888
NSAIDs	404	55.04%	410	55.86%	0.753	651	62.36%	685	65.61%	0.121
COX-2 inhibitors	27	3.68%	33	4.50%	0.429	26	2.49%	20	1.92%	0.371
Steroids	134	18.26%	145	19.75%	0.464	223	21.36%	240	22.99%	0.371
Clopidogrel	5	0.68%	2	0.27%	0.256	11	1.05%	12	1.15%	0.834
Ticlopidine	3	0.41%	4	0.54%	0.705	10	0.96%	10	0.96%	1.000
Warfarin	6	0.82%	7	0.95%	0.781	10	0.96%	12	1.15%	0.668
**During hospitalization**										
Use of PPI/H_2_RA	650	88.56%	631	85.97%	0.137	874	83.72%	896	85.82%	0.180
Infection	146	19.89%	139	18.94%	0.644	184	17.62%	202	19.35%	0.310
**Indicator of severity of bleeding**										
Coagulation defects	8	1.09%	1	0.14%	0.019	6	0.57%	0	0.00%	0.014
Need for endoscopic Intervention	261	35.56%	192	26.16%	< .0001	242	23.18%	248	23.75%	0.757
Shock	30	4.09%	25	3.41%	0.492	23	2.20%	5	0.48%	0.770
Requirement for mechanical ventilation	41	5.59%	53	7.22%	0.201	70	6.70%	71	6.80%	0.931
Malnutrition	3	0.41%	4	0.54%	0.705	4	0.38%	7	0.67%	0.364

**Abbreviations:** NSAIDs, nonsteroidal anti-inflammatory drugs; COX-2 inhibitors, cyclooxygenase-2 inhibitors; PPI/H_**2**_RA, proton pump inhibitors/histamine type 2 receptor antagonists.

*Individual disease conditions in the Charlson Comorbid Index were accounted to generate a propensity score for each patient. Data were not presented if sample size is 0 (e.g. HIV, liver diseases).

Continuous data are presented as mean ± standard deviation, and categorical data as n and %. Pearson’s chi-square or Fisher’s exact test was used to examine categorical data, and 2-sample t tests were conducted for continuous data.

Table 2 shows the outcome of the matched cohort. During the index hospitalization in the matched cohort, 91 (12.4%) out of 734 cirrhotic patients experienced recurrent PUB and were treated using transarterial embolization (55/91, 60.4%) more often than the matched controls (both P < 0.0001). Moreover, 70 (6.7%) out of 1044 chronic hepatitis patients experienced recurrent PUB but did not significantly differ from the matched controls (6.7% vs. 5.56%, P = 0.2736). Chronic hepatitis and matched controls groups received higher frequency of surgical intervention to control rebleeding than embolization group. After hospital discharge, the recurrent PUB was not significantly different among the matched controls (cirrhosis vs. matched control: 5.68% vs. 4.34%, P = 0.2449; chronic hepatitis vs. control: 5.5% vs. 4.94%, P = 0.5693). However, the mortality was higher in the cirrhosis and chronic hepatitis groups than that in the matched controls during the long-term follow-up (cirrhosis vs. matched controls: 24.15% vs. 19.16%, P = 0.0225; chronic hepatitis vs. matched controls: 15.72% vs. 12.89%, P = 0.0672). The hospitalization cost (US$ 1881 ± 2942 vs. US$ 1539 ± 3841, P = 0.0172) and the length of hospital stay (11.1 ± 10.9 vs. 9.4 ± 11.7, P = 0.0002) were significantly higher in the cirrhosis group than those in the matched controls (Table 2).

**Table 2:** Outcomes and rebleeding management of study cohort (propensity score-matched cohort).

Outcomes	Cirrhosis (n = 734)		Matched controls (n = 734)		P-value	Chronic hepatitis (n = 1044)		Matched controls (n = 1044)		P-value
**During hospitalization**										
Rebleeding	91	12.40%	40	5.45%	< .0001	70	6.70%	58	5.56%	0.274
Surgery	34	4.63%	31	4.22%	0.704	42	4.02%	47	4.50%	0.588
TAE	55	7.49%	7	0.95%	< .0001	27	2.59%	7	0.67%	0.001
Repeat Endoscopy	4	0.54%	3	0.41%	0.705	2	0.19%	7	0.67%	0.094
Death	30	4.09%	19	2.59%	0.110	26	2.49%	22	2.11%	0.559
Total cost ($USD)	1881.1 ± 2942.1		1538.8± 3840.8		0.017	1223.6 ± 2803.4		1419.6 ± 4209.4		0.211
Length of Stay (days)	11.1±10.9		9.4±11.7		0.0002	8.3±8.9		9.2±16.5		0.098
**After hospitalization**	Cirrhosis (n = 704)		Controls (n = 715)		P-value	Chronic hepatitis(n = 1018)		Controls (n = 1022)		P-value
Rebleeding^a^	40	5.68%	31	4.34%	0.224	56	5.50%	51	4.99%	0.605
Death	170	24.15%	137	19.16%	0.023	160	15.72%	133	13.01%	0.082

**Abbreviations**: TAE:, transarterial embolization.

a. Rebleeding after hospital discharge defined as peptic ulcer bleeding proven endoscopically and requiring endoscopic intervention.

Continuous data are presented as mean ± standard deviation, and categorical data as n and %. Pearson’s chi-square or Fisher’s exact test was used to examine categorical data, and 2-sample t test was conducted for continuous data.

### Independent risk factors of recurrent PUB in the matched cohort during a 12-year follow-up period

**Fig 2:**
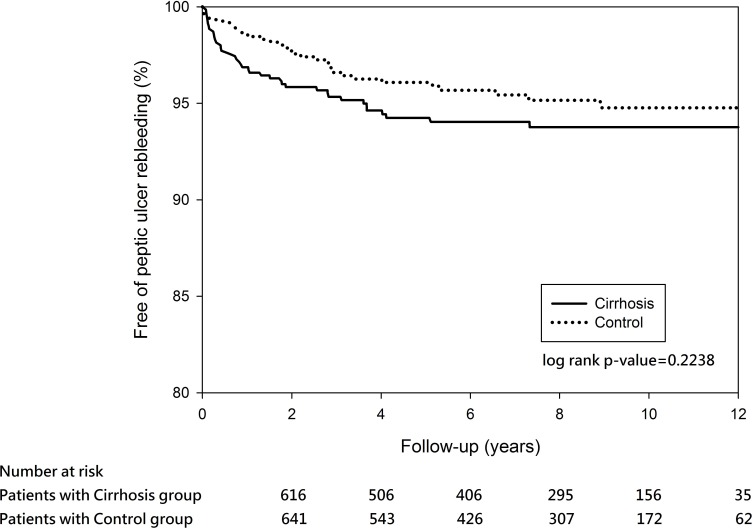
Kaplan–Meier estimates of outcomes in the propensity score-matched cohort for peptic ulcer rebleeding event-free survival among patients with cirrhosis matched with controls (P = 0.2238).

In the propensity score-matched cohort, 40 (5.68%) out of the 704 cirrhosis patients experienced recurrent PUB within the 12-year observation (Table 2). The cumulative rebleeding-free rates at 5 years in the matched cirrhosis and controls were 94.25% and 96.09%, respectively. Log-rank test revealed no significant difference between the two groups over time (P = 0.2238) (Fig 2). In the Cox regression model, the independent risk factors of recurrent PUB after discharge were prior peptic ulcer history, pulmonary disease, and use of steroids in the cirrhosis matched cohort (Table 3).

**Table 3:** Factors associated with rebleeding in patients with cirrhosis (propensity score-matched cohort).

Variable	During hospitalization			After hospitalization		
	OR	95% CI	P-value	HR	95% CI	P-value
Liver cirrhosis (vs. matched controls)	2.62	1.74–3.92	< .0001	1.29	0.80–2.09	0.291
Age	1.00	0.98–1.01	0.605	1.01	0.99–1.03	0.380
Female gender	0.92	0.59–1.44	0.712	0.76	0.42–1.37	0.353
**Comorbid conditions**						
Acute myocardial infarction	1.48	0.15–14.33	0.734	1.82	0.21–16.16	0.591
Congestive heart failure	0.78	0.33–1.87	0.581	1.02	0.35–2.96	0.971
Peripheral vascular disease	0.26	0.03–2.38	0.231	1.94	0.45–8.30	0.373
Cerebral vascular accidents	0.57	0.24–1.33	0.191	0.99	0.41–2.35	0.974
Dementia	0.35	0.04–2.72	0.314	0.85	0.19–3.78	0.833
Pulmonary disease	1.01	0.60–1.69	0.982	0.45	0.22–0.96	0.038
Connective tissue disorder	2.70	0.67–10.83	0.161	0		
Peptic ulcer	1.44	0.98–2.12	0.062	0.96	0.59–1.56	0.858
Diabetes	0.90	0.54–1.48	0.674	0.84	0.43–1.64	0.614
Diabetes complications	1.09	0.51–2.34	0.826	0.72	0.23–2.23	0.570
Paraplegia	0.38	0.04–3.91	0.418	1.64	0.35–7.76	0.534
Renal disease	0.88	0.47–1.66	0.695	1.06	0.48–2.31	0.891
Cancer	2.05	1.35–3.13	0.001	0.73	0.40–1.35	0.318
Metastatic cancer	1.08	0.48–2.40	0.857	0.28	0.04–2.13	0.216
Prior ulcer history (≤180 days)	1.42	0.81–2.48	0.218	1.97	1.07–3.61	0.029
**Prior use of ulcerogenic drugs (≤90 days)**						
Aspirin	0.83	0.43–1.60	0.578	1.27	0.59–2.74	0.545
NSAIDs	1.15	0.76–1.73	0.518	1.34	0.80–2.26	0.269
COX-2 inhibitors	0.47	0.14–1.62	0.233	0.66	0.16–2.82	0.576
Steroids	1.35	0.85–2.15	0.199	2.21	1.27–3.85	0.005
Clopidogrel	1.20	0.12–12.41	0.880	6.21	0.72–53.5	0.096
**During hospitalization**						
Use of PPI/H_2_RA	1.39	0.72–2.69	0.322	1.04	0.51–2.11	0.925
Infection	1.50	1.00–2.25	0.049	0.49	0.28–0.83	0.009
**Indicator of severity of bleeding**						
Coagulation defects	0.94	0.10–8.43	0.955	1.98	0.26–15.31	0.512
Need for endoscopic intervention	4.32	2.46–7.58	< .0001	0		
Shock	2.24	1.01–4.96	0.047	0.36	0.05–2.68	0.320
Requirement for mechanical ventilation	1.11	0.73–1.70	0.620	1.29	0.77–2.18	0.331
Malnutrition	2.71	0.29–25.65	0.386	0		

**Abbreviations:** OR, odds ratio; CI, confidence interval; HR, hazard ratio; NSAIDs, nonsteroidal anti-inflammatory drugs; COX-2 inhibitors, cyclooxygenase-2 inhibitors; PPI/H_2_RA, proton pump inhibitors/histamine type 2 receptor antagonists.

The effect of cirrhosis on rebleeding was compared with propensity score-matched patients in the control group with 1:1 ratio. Patients with cirrhosis were defined withde*c*ompensated and compensated cirrhosis.

**Fig 3:**
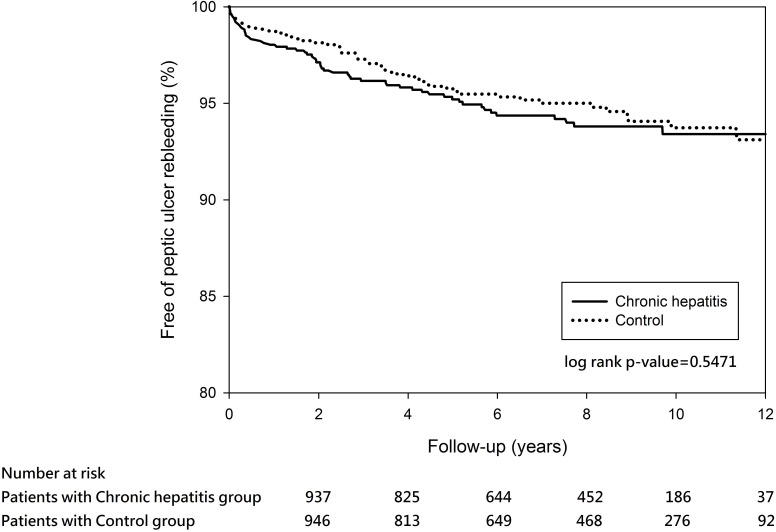
Kaplan–Meier estimates of outcomes in the propensity score-matched cohort for peptic ulcer rebleeding event-free survival among patients with chronic hepatitis matched with controls (P = 0.5471).

A total of 56 (5.5%) out of the 1018 propensity score-matched chronic hepatitis patients and 51(4.99%) out of the 1022 matched controls experienced recurrent PUB within the 12-year observation (Table 2). The cumulative rebleeding-free rates at 5 years in the matched chronic hepatitis and matched controls were 95.21% and 95.75%, respectively. Log-rank test revealed no significant difference between the two groups over time (P = 0.5471) (Fig 3). The independent risk factors of recurrent PUB after discharge were pulmonary disease and use of PPI/H2RA during index hospitalization after adjustment for other covariates (Table 4).

**Table 4:** Factors associated with rebleeding in patients with chronic hepatitis (propensity score-matched cohort).

Variable	During hospitalization			After hospitalization		
	OR	95% CI	P-value	HR	95% CI	P-value
**Chronic hepatitis (vs. matched controls)**	1.24	0.86–1.79	0.251	1.15	0.78–1.69	0.474
Age	1.01	1.00–1.02	0.173	1.01	1.00–1.02	0.202
Female gender	0.64	0.41–1.00	0.049	0.93	0.61–1.43	0.750
**Comorbid conditions**						
Congestive heart failure	0.72	0.33–1.56	0.400	1.51	0.77–2.95	0.229
Cerebral vascular accidents	0.58	0.30–1.10	0.095	0.92	0.50–1.71	0.802
Dementia	0.31	0.04–2.38	0.262	1.44	0.44–4.73	0.552
Pulmonary disease	0.68	0.41–1.14	0.146	0.51	0.29–0.91	0.023
Connective tissue disorder	1.01	0.29–3.58	0.987	0.40	0.05–2.91	0.364
Peptic ulcer	1.17	0.81–1.69	0.411	1.24	0.84–1.83	0.270
Diabetes	0.84	0.51–1.37	0.475	1.17	0.72–1.90	0.529
Diabetes complications	0.80	0.30–2.15	0.658	0.14	0.02–1.04	0.054
Paraplegia	0			1.49	0.35–6.40	0.595
Renal disease	1.24	0.69–2.22	0.465	1.13	0.59–2.14	0.714
Cancer	1.13	0.62–2.07	0.695	0.94	0.46–1.95	0.872
Metastatic cancer	0.35	0.04–2.79	0.321	2.02	0.54–7.53	0.296
Prior ulcer history (≤180 days)	0.94	0.41–2.16	0.891	1.84	0.96–3.52	0.067
**Prior use of ulcerogenic drugs (≤90 days)**						
Aspirin	0.38	0.17–0.85	0.019	0.88	0.46–1.68	0.688
NSAIDs	1.04	0.69–1.57	0.859	1.08	0.70–1.66	0.729
COX-2 inhibitors	0.72	0.16–3.19	0.664	0.31	0.04–2.31	0.254
Steroids	1.29	0.82–2.02	0.278	1.59	1.00–2.51	0.048
Clopidogrel	2.15	0.45–10.16	0.335	1.03	0.14–7.76	0.976
Ticlopidine	0.78	0.33–1.8	0.560	2.17	0.51–9.32	0.297
Warfarin	0.93	0.12–7.22	0.943	2.55	0.66–9.93	0.176
**During hospitalization**						
Use of PPI/H_2_RA	1.22	0.69–2.14	0.494	2.69	1.24–5.84	0.012
Infection	1.43	0.97–2.10	0.071	0.93	0.61–1.40	0.711
**Indicator of severity of bleeding**						
Coagulation defects	1.07	0.27–4.29	0.928	3.12	0.31–31.32	0.333
Need for endoscopicintervention	2.73	1.63–4.58	< .001	0		
Shock	1.60	0.63–4.07	0.325	0.37	0.05–2.69	0.326
Requirement for mechanical ventilation	1.77	1.17–2.70	0.007	1.03	0.65–1.64	0.888

**Abbreviations:** OR, odds ratio; CI, confidence interval; HR, hazard ratio; NSAIDs, nonsteroidal anti-inflammatory drugs; COX-2 inhibitors, cyclooxygenase-2 inhibitors; PPI/H_2_RA, proton pump inhibitors/histamine type 2 receptor antagonists.

The effect of chronic hepatitis (without cirrhosis) on rebleeding was compared with propensity score-matched patients in the control group with 1:1 ratio.

During the 12-year follow-up period, the adjusted HR of recurrent PUB after discharge was 1.29 (95% CI, 0.8–2.09; P = 0.291) in the cirrhotic patients matched analysis, and 1.15 (95% CI, 0.78–1.69; P = 0.474) in the chronic hepatitis patients matched analysis.

### Independent risk factors of death in the matched cohort during a 12-year follow-up period

**Fig 4:**
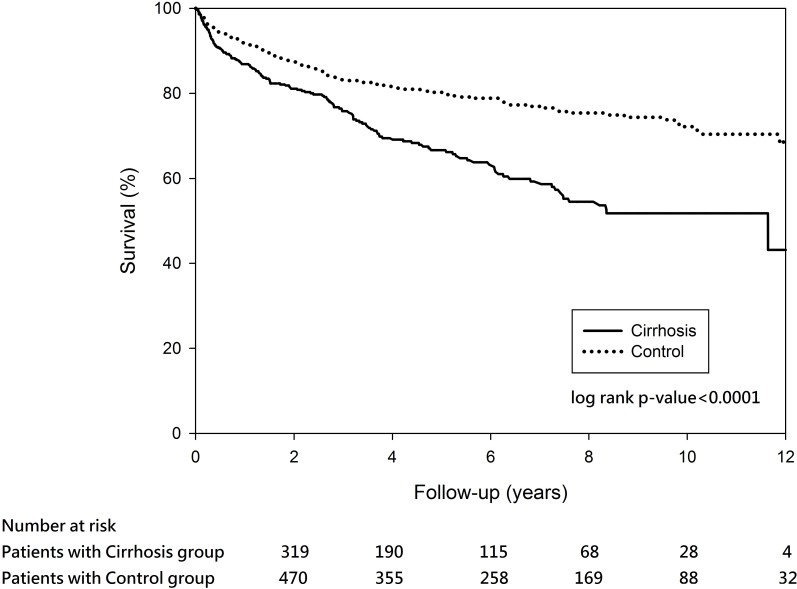
Survival probability among patients with cirrhosis matched with controls (P < 0.0001).

In the propensity score-matched analyses, there were 30 (4.09%) of the 734 cirrhotic patients died during hospitalization, and 170 (24.15%) of the 704 cirrhotic patients died within the 12-year observation. Cirrhosis group exhibited higher in-hospital mortality than the matched controls but the difference was not significant (4.09% vs. 2.59%, P = 0.11). The survival rates in the matched cirrhosis groups were 86.88% at year 1, 81.1% at year 2, 75.82% at year 3, 69.4% at year 4, and 66.65% at year 5. The survival rates in the cirrhosis-matched controls were 91.91% at year 1, 87.58% at year 2, 83.22% at year 3, 81.46% at year 4, and 80.25% at year 5. Log-rank test revealed a statistically significant difference in survival between the cirrhosis and its matched controls over time (P <0.0001) (Fig 4). Factors significantly associated with increased risk of in-hospital mortality were older age, dementia, cancers, coagulation defects, and requirement for mechanical ventilation in the cirrhotic patients matched analysis (Table 5). Liver cirrhosis, older age, dementia, cancer, and requirement for mechanical ventilation during index hospitalization were significantly associated with additional risk of long-term mortality (Table 5).

**Table 5:** Factors associated with all-cause mortality in patients with cirrhosis(propensity score-matched cohort).

Variable	During hospitalization			After hospitalization		
	OR	95% CI	P-value	HR	95% CI	P-value
Liver cirrhosis (vs. matched controls)	1.70	0.90–3.20	0.101	2.04	1.61–2.58	< .0001
Age	1.04	1.01–1.07	0.006	1.02	1.02–1.03	< .0001
Female gender	0.92	0.46–1.83	0.805	0.83	0.63–1.08	0.165
**Comorbid conditions**						
Acute myocardial infarction	0			0.54	0.07–3.97	0.548
Congestive heart failure	0.96	0.35–2.66	0.943	1.20	0.78–1.85	0.412
Peripheral vascular disease	2.77	0.72–10.68	0.140	0.52	0.21–1.30	0.163	
Cerebral vascular accidents	0.79	0.29–2.19	0.654	1.02	0.71–1.47	0.901
Dementia	2.19	0.62–7.79	0.225	2.45	1.42–4.24	0.001
Pulmonary disease	0.84	0.39–1.84	0.668	0.86	0.64–1.16	0.335
Connective tissue disorder	2.06	0.20–21.74	0.547	1.18	0.46–2.99	0.735
Peptic ulcer	0.74	0.39–1.41	0.354	1.02	0.80–1.29	0.886
Diabetes	1.22	0.58–2.54	0.605	1.60	1.21–2.12	0.001
Diabetes complications	2.02	0.81–5.04	0.131	1.30	0.86–1.95	0.213
Paraplegia	2.81	0.46–17.00	0.261	1.04	0.37–2.95	0.937
Renal disease	1.33	0.58–3.07	0.497	0.90	0.62–1.31	0.581
Cancer	2.70	1.36–5.34	0.005	2.64	2.01–3.45	< .0001
Metastatic cancer	2.86	1.01–8.07	0.045	1.56	0.83–2.94	0.166
Prior HP therapy(≤180 days)	2.38	0.38–15.04	0.355	1.40	0.50–3.94	0.520
**Prior ulcer history (≤180 days)**	0.27	0.06–1.20	0.085	1.10	0.79–1.54	0.569
**Prior use of ulcerogenic drugs (≤90 days)**						
Aspirin	0.78	0.24–2.51	0.678	1.00	0.67–1.49	0.988
NSAIDs	0.68	0.35–1.34	0.269	0.79	0.61–1.01	0.063
COX-2 inhibitors	2.26	0.75–6.81	0.149	0.61	0.29–1.31	0.207
Steroids	0.84	0.36–1.96	0.683	1.22	0.90–1.66	0.195
Clopidogrel	1.19	0.04–38.82	0.921	1.03	0.24–4.34	0.969
Ticlopidine	1.44	0.04–48.07	0.839	1.71	0.51–5.75	0.390
Warfarin	1.83	0.20–16.56	0.593	1.10	0.79–1.54	0.569
**During hospitalization**						
Use of PPI/H_2_RA	2.38	0.68–8.33	0.174	0.89	0.65–1.22	0.462
Infection	0.63	0.32–1.26	0.191	0.97	0.87–1.08	0.580
**Indicator of severity of bleeding**						
Coagulation defects	2.94	1.12–7.76	0.029	1.82	0.44–7.52	0.411
Need for endoscopicintervention	2.12	0.78–5.78	0.140	1.15	0.97–1.36	0.118
Shock	1.33	0.41–4.29	0.637	1.02	0.51–2.05	0.955
Requirement for mechanical ventilation	2.56	1.34–4.88	0.004	3.26	2.54–4.17	< .0001

**Abbreviations:** OR, odds ratio; CI, confidence interval; HR, hazard ratio; NSAIDs, nonsteroidal anti-inflammatory drugs; COX-2 inhibitors, cyclooxygenase-2 inhibitors; PPI/H_2_RA, proton pump inhibitors/histamine type 2 receptor antagonists.

The effect of cirrhosis on all-cause mortality was compared with propensity score-matched patients in the control group with 1:1 ratio. Patients with cirrhosis were defined withde*c*ompensated and compensated cirrhosis.

**Fig 5:**
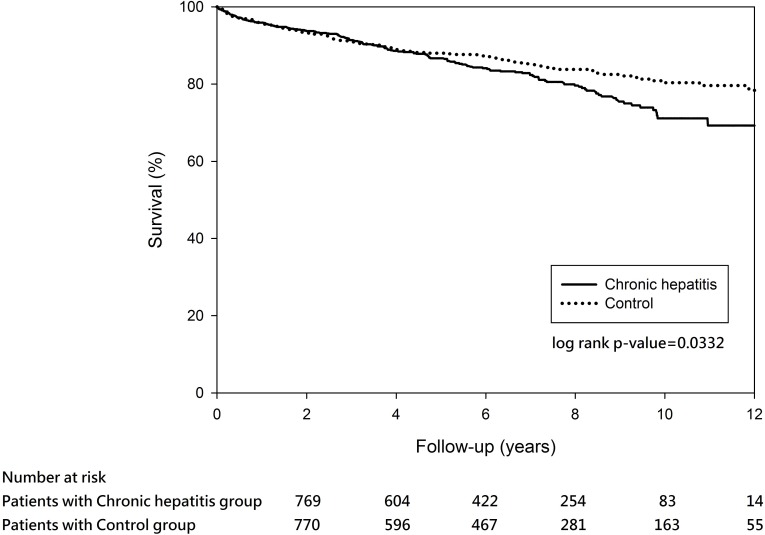
Survival probability among patients with chronic hepatitis matched with controls (P = 0.0332).

On the other hand, 26 (2.49%) out of the 1044 chronic hepatitis patients died during hospitalization and 160 (15.72%) out of the 1018 cirrhosis patients died within the 12-year observation. The in-hospital mortality between chronic hepatitis and its matched controls was similar (2.49% vs. 2.11%, P = 0.9486). The survival rates in the chronic hepatitis group were 95.93% at year 1, 93.75% at year 2, 91.41% at year 3, 88.60% at year 4, and 86.70% at year 5. The survival rates in the chronic hepatitis matched controls were 95.86% at year 1, 93.46% at year 2, 91.07% at year 3, 88.95% at year 4, and 88.02% at year 5. Log-rank test revealed a statistically significant difference in survival rates between the chronic hepatitis and its matched controls groups over time (P <0.0332) (Fig 5). In the chronic hepatitis matched cohort, chronic hepatitis, older age, cancer, and requirement for mechanical ventilation during index hospitalization significantly increased risk of mortality during index hospitalization and after discharge (Table 6).

**Table 6:** Factors associated with all-cause mortality in patients with chronic hepatitis(propensity score-matched cohort).

Variable	During hospitalization			After hospitalization		
	OR	95% CI	P-value	HR	95% CI	P-value
Chronic hepatitis (vs. matched controls)	2.45	1.13–5.31	0.023	1.39	1.10–1.76	0.007
Age	1.04	1.00–1.07	0.034	1.03	1.02–1.04	< .0001
Female gender	0.94	0.42–2.12	0.879	0.76	0.59–0.99	0.040
**Comorbid conditions**						
Acute myocardial infarction	2.63	0.42–16.42	0.302	0.40	0.09–1.76	0.222
Congestive heart failure	0.51	0.13–1.98	0.328	1.44	1.00–2.09	0.051	
Peripheral vascular disease				1.93	0.61–6.13	0.263
Cerebral vascular accidents	1.13	0.46–2.78	0.798	1.11	0.81–1.51	0.514
Dementia	3.13	0.91–10.81	0.072	1.48	0.86–2.55	0.160
Pulmonary disease	1.56	0.70–3.47	0.272	0.99	0.75–1.32	0.969
Connective tissue disorder				1.03	0.45–2.38	0.943
Peptic ulcer	0.46	0.21–1.00	0.049	0.89	0.70–1.13	0.324
Diabetes	1.33	0.58–3.06	0.497	1.60	1.21–2.10	0.001
Diabetes complications	2.06	0.56–7.59	0.279	1.21	0.74–1.97	0.457
Paraplegia	1.10	0.10–11.61	0.938	0.77	0.24–2.50	0.668
Renal disease	0.98	0.35–2.76	0.973	1.09	0.76–1.57	0.647
Cancer	5.99	2.57–13.93	< .0001	3.26	2.30–4.63	< .0001
Metastatic cancer	3.66	1.08–12.42	0.038	1.25	0.30–5.28	0.764
Prior ulcer history (≤180 days)	0.98	0.26–3.65	0.973	0.92	0.58–1.46	0.734
**Prior use of ulcerogenic drugs (≤90 days)**						
Aspirin	0.68	0.17–2.66	0.577	1.00	0.71–1.42	0.999
NSAIDs	0.48	0.23–1.02	0.058	0.96	0.74–1.25	0.762
COX-2 inhibitors	0.91	0.69–1.21	0.519	1.24	0.63–2.43	0.537
Steroids	0.94	0.39–2.27	0.884	0.91	0.69–1.22	0.530
Clopidogrel	3.67	0.40–33.29	0.248	1.24	0.45–3.43	0.681
Ticlopidine	0.68	0.27–1.73	0.422	0.84	0.33–2.11	0.708
Warfarin	1.24	0.10–14.83	0.865	1.22	0.53–2.82	0.647
**During hospitalization**						
Use of PPI/H2RA	0.96	0.34–2.70	0.935	1.29	0.89–1.88	0.183
Infection	1.37	0.65–2.89	0.411	1.06	0.82–1.36	0.663
**Indicator of severity of bleeding**						
Coagulation defects	1.22	0.97–1.54	0.093	3.55	0.50–25.32	0.207
Need for endoscopic intervention	1.50	0.40–5.57	0.548	1.16	0.94–1.43	0.179
Shock	2.41	0.64–9.12	0.195	0.55	0.19–1.61	0.273
Requirement for mechanical ventilation	3.64	1.74–7.62	0.001	5.78	4.45–7.50	< .0001

**Abbreviations:** OR, odds ratio; CI, confidence interval; HR, hazard ratio; NSAIDs, nonsteroidal anti-inflammatory drugs; COX-2 inhibitors, cyclooxygenase-2 inhibitors; PPI/H_2_RA, proton pump inhibitors/histamine type 2 receptor antagonists.

The effect of chronic hepatitis (without cirrhosis) was compared with propensity score-matched patients in the control group with 1:1 ratio.

## Discussion

No long-term follow-up studies of a large cohort of patients have been conducted to evaluate the occurrence of recurrent PUB and mortality in cirrhotic patients after discharge. Moreover, few *studie*s have compared the outcomes among patients with cirrhosis or chronic hepatitis and patients without liver disease. Given the lack of population-based studies, we investigated the effect of cirrhosis on PUB. The current nationwide population-based longitudinal cohort study compared the occurrence of recurrent PUB and survival among cirrhotic patients, chronic hepatitis patients, and matched controls after a follow-up period of up to 12 years. This study utilized Taiwan’s NHIRD. The cirrhotic patients with PUB were at increased risk for recurrent bleeding during hospitalization and exhibited higher mortality in the 12-year follow-up after discharge. During hospitalization, the patients with cirrhosis were at 2.62-fold higher risk of developing recurrent PUB (95% CI: 1.74–3.92) after confounding adjustment. In addition, cirrhosis was associated with increased hospital costs and length of stay.

The in-hospital recurrent PUB rate of 12.4% in the cirrhosis group was consistent with previous studies [4, 13, 14]. Interestingly, the cirrhotic patients with recurrent PUB were less frequently operated compared with chronic hepatitis patients and matched controls in the current study. This result may be attributed to the occurrence of more complications, such as perioperative adverse events, increasing mortality rate, length of stay, and total hospital costs in cirrhotic patients [15]. Consequently, most doctors chose non-surgical treatment for PUB, such as arterial embolization, which is recommended as the salvage treatment of choice for high-surgical-risk cirrhotic patients [4].

The long-term course of peptic ulcer rebleeding in patients with liver cirrhosis is not well established. It is possibly due to aged patients and concomitant comorbidities in this cirrhotic cohort, resulting in poor long-term prognosis. Identification of severe peptic ulcer rebleeding is necessary, and endoscopic intervention is commonly required in these critically ill patients. Hsu at al. [16] reported that liver cirrhosis and older age are independent risk factors for long-term peptic ulcer rebleeding. However, this finding is not consistent with the result in the present study. The discrepancy may be attributed to the difference in the *definition* of long-term rebleeding. Hsu et al. defined recurrent PUB as re-hospitalization with a primary diagnosis of PUB after the index bleeding episode during the study period. Our study strictly defined long-term rebleeding as a re-hospitalization event with a primary diagnosis of PUB (codes: 531.0, 531.2, 531.4, 531.6, 532.0, 532.2, 532.4, 532.6, 533.0, 533.2, 533.4, and 533.6) that requires endoscopic intervention (billing code: 47043B). Although liver cirrhosis increased the risk of recurrent PUB during hospitalization (OR, 2.62, 95% CI, 1.74–3.92; P < .0001), the significance was not observed at follow-up (HR, 1.29, 95% CI 0.8–2.09; P = 0.291). The 12-year cumulative incidence of recurrent bleeding requiring re-hospitalization and endoscopic therapy was 5.68% in the cirrhotic cohort and 4.34% in matched controls (P = 0.224).

The mortality of PUB remains as high as 10% despite the recent advances on the treatment modalities [4, 14–16]. The current study showed lower in-hospital mortality of 4.09% compared with the previously published series [4, 14–18]. Nevertheless, the survival rate in the cirrhosis group was the lowest among the three groups during the 12-year follow-up after discharge. Our study has clearly shown the increased long-term mortality in both cirrhotic and non-cirrhotic patients with chronic hepatitis. If certain predictors could be identified to predict mortality in patients with PUB, this could potentially develop the best therapeutic strategies to optimize care and reduce mortality. No evidence is available in literature that shows that most PUB-associated deaths in patients with cirrhosis are a direct result of PUB bleeding itself. Instead, comorbidity was more relevant to the cause of death in cirrhotic patients with PUB. This finding could explain the high mortality rate despite the recent advancement in endoscopic hemostasis for PUB [16–19].

However, the need for endoscopic intervention did not reach statistically significant associated with mortality in both cirrhosis and chronic hepatitis matched comparisons in the present study, which was similarly reported by Gonzalez-Gonzalez et al. [3]. The fact emphasizes that mortality in cirrhotic patients with PUB was associated with multiple organ failure or cardiopulmonary conditions, suggesting that concomitant co-morbidities played a fundamental role for death.

For instance, accumulated evidence suggests that the presence of diabetes is associated with worse outcomes of liver diseases [20–23]. Diabetic patients have high incidences of coronary heart disease, chronic renal disease, and cirrhosis, as well as peptic ulcer and use of antiplatelet agents [24–27]. Cox regression analysis in the present study showed that diabetes is an independent risk factor (adjusted HR: 1.6 in both cirrhosis and chronic hepatitis matched cohort analyses) of long-term morality. The increased mortality rate in cirrhotic and chronic hepatitis patients with diabetes is not only due to the complications of diabetes itself but also to the increased risk of hepatic failure [21–23]. Comorbidity is strongly associated with mortality in PUB patients, particularly cirrhosis. Thus, identification and treatment of comorbid diseases concomitantly in cirrhotic patients with PUB are the first priority. The need for more comprehensive actions to provide a strong support for the major organ systems when treating these patients is an extremely important issue [28].

Several studies have suggested that COPD is associated with an increased risk of rebleeding in patients with PUB [29–31]. Some reasons that may explain why COPD increases the risk of PUB are as follows. First, the major cause is hypersecretion of gastric acid and decreased mucosa defense mechanism induced by hypoxemia or hypercapnia [32, 33]. Second, COPD patients are often treated with oral steroids, which seem to increase the risk of ulcer bleeding. After matching, use of steroids was an independent predictor of long-term peptic ulcer re-bleeding in both cirrhosis and chronic hepatitis matched analyses. Additional potential confounders, such as *Helicobacter pylori* infection status (small number of cases in the present study) and lifestyle factors (e.g., smoking, alcohol consumption), should be considered in future research to clarify the role of COPD in patients with ulcer bleeding.

The NHIRD has all the attributes to investigate multiple factors, but several limitations also exist. First, this observational retrospective cohort study was based on hospitalized patients with PUB in which the diagnosis and identification of comorbidity were dependent on the accuracy of coding procedures. This method could result in selection biases; thus, caution must be exercised during interpretation of the results. Second, *Rockall*score for *risk stratification* in patients with PUB could not be calculated because endoscopic details, such as s*tigmata of hemorrhage in* bleeding peptic ulcers, were not recorded in the NHIRD. Therefore, we assessed other factors, such as the presence of shock, need of endoscopic intervention, and requirement for mechanical ventilation support, to replace the indicators of severity of bleeding. Third, no information on lifestyle factors, such as alcohol consumption, smoking, and malnutrition, were available. These lifestyle factors have been reported to be prognostic markers among cirrhotic patients [34, 35]. These factors could have confounded the present findings if they were prevalent among patients with comorbidities. Fourth, given the reimbursement restrictions of the NHI program and the resultant ceiling effect, somemedical services could not be refunded in the event of repeated endoscopic treatment for rebleeding ulcers during the same hospitalization. Repeated endoscopic interventions may not be reimbursed by the NHI program and therefore not recorded in the procedure coding system. This may have underestimated the frequency of endoscopic interventions (billing code: 47043B) in the hospitalized cohort obtained from the NHID. Lastly, *H*. *pylori* infection is an important risk factor for ulcer bleeding; however, we could not obtain sufficient data for the prevalence of *H*. *pylori* in the NHIRD. Despite these limitations, this study has several strengths and clinical implications. A major strength of our study is the age- and gender-matched cohort design. Other strengths include the long-term follow-up and adequate adjustments for baseline comorbidities, severity of bleeding ulcers, and drug uses in data analysis. Furthermore, we also analyzed non-cirrhotic chronic hepatitis patients and performed PSM analysis to select patients without liver disease as matched controls. The use of data from a nationwide database minimizes the potential for possible bias that may be observed in single-center studies. The database serves as a powerful tool to study significant effects of cirrhosis on PUB outcomes.

In conclusion, liver cirrhosis increased health care expenses in patients with PUB and these patients exhibited higher recurrent bleeding rate than non-cirrhotic patients during hospitalization. Cirrhosis and chronic hepatitis were independently associated with an increased long-term mortality when compared with the patients without liver disease.

## Supporting Information

S1 Table This table provides data on the characteristics of the study cohort among patients with cirrhosis, chronic hepatitis, and those who were in the control group before propensity score-matched. (DOC)
