# Supplementary material for: Risk of Rebleeding and Mortality in Cirrhotic Patients with Peptic Ulcer Bleeding: A 12-Year Nationwide Cohort Study
Source: PLoS One. 2017 Jan 12;12(1):e0168918. doi: 10.1371/journal.pone.0168918 (PMC5233423; doi:10.1371/journal.pone.0168918)
Supplement: S1 Table — (DOC) [file pone.0168918.s001.doc]

**Supplemental table 1 .Characteristics of the study cohort among patients with cirrhosis, chronic hepatitis, and those who were in the control group (n=15,575)**

|  | Cirrhosis  (n = 737) | | Chronic hepatitis  (n = 1044) | | Control  (n = 13794) | |
| --- | --- | --- | --- | --- | --- | --- |
| Age, years | 60.97 ±14.60* | | 59.35 ±16.16† | | 63.34 ±17.17 | |
| Male gender | 520 | 70.56%* | 732 | 70.11%† | 9064 | 65.71% |
| Charlson comorbid index score (<=365 days before index date) | 3.62±2.24 | | 2.50±1.72† | | 1.22±1.62 | |
| Acute myocardial infarction | 6 | 0.81% | 15 | 1.44% | 186 | 1.35% |
| Congestive heart failure | 59 | 8.01%* | 68 | 6.51% | 805 | 5.84% |
| Peripheral vascular disease | 15 | 2.04%* | 7 | 0.67% | 164 | 1.19% |
| Cerebral vascular accid | 72 | 9.77%* | 146 | 13.98% | 1953 | 14.16% |
| Dementia | 21 | 2.85% | 26 | 2.49% | 438 | 3.18% |
| Pulmonary disease | 146 | 19.81%* | 210 | 20.11%† | 2197 | 15.93% |
| Connective tissue disorder | 10 | 1.36% | 24 | 2.30%† | 169 | 1.23% |
| Diabetes | 168 | 22.80%* | 220 | 21.07%† | 1845 | 13.38% |
| Diabetes complications | 66 | 8.96%* | 50 | 4.79% | 610 | 4.42% |
| Paraplegia | 12 | 1.63% | 16 | 1.53% | 173 | 1.25% |
| Renal disease | 92 | 12.48%* | 102 | 9.77%† | 1037 | 7.52% |
| Cancer | 213 | 28.90%* | 106 | 10.15%† | 760 | 5.51% |
| Metastatic cancer | 30 | 4.07%* | 19 | 1.82% | 186 | 1.35% |
| HIV | 0 | 0.00% | 0 | 0.00% | 5 | 0.04% |
| Prior ulcer history (<=180days) | 83 | 11.26%* | 60 | 5.75%† | 566 | 4.10% |
| Prior HP history (<=180 days) | 9 | 1.22%* | 4 | 0.38% | 39 | 0.28% |
| Prior uses of ulcerogenic drugs (prior <=90 days) |  |  |  |  |  |  |
| ASA | 71 | 9.63% | 115 | 11.02%† | 1236 | 8.96% |
| NSAIDs | 406 | 55.09% | 651 | 62.36%† | 7211 | 52.28% |
| Steroids | 134 | 18.18% | 223 | 21.36%† | 2443 | 17.71% |
| Clopidogrel | 5 | 0.68% | 11 | 1.05% | 215 | 1.56% |
| Ticlopidine | 3 | 0.41%* | 10 | 0.96% | 172 | 1.25% |
| Warfarin | 6 | 0.81% | 10 | 0.96% | 170 | 1.23% |
| **During hospitalization** |  |  |  |  |  |  |
| Need for endoscopic intervention | 42 | 5.70% | 70 | 6.70% | 937 | 6.79% |
| PPI/H_2_RA administration | 653 | 88.60%* | 874 | 83.72% | 11781 | 85.41% |
| Infection | 146 | 19.81% | 184 | 17.62%† | 2948 | 21.37% |
| Caoagulation defects | 8 | 1.09% | 6 | 0.57% | 42 | 0.30% |
| Shock | 30 | 4.07% | 23 | 2.20%† | 465 | 3.37% |
| Requirement for mechanical  ventilation | 263 | 35.69%* | 242 | 23.18% | 3572 | 25.90% |
| Malnutrition | 3 | 0.41% | 4 | 0.38% | 64 | 0.46% |

**Abbreviations:** NSAIDs, nonsteroidal anti-inflammatory drugs;PPI/H2-blockers, promton pump inhibitors/H_2_RA, histamine type 2 receptor antagonists

Continuous data are as mean ± standard deviation; categorical data are as n and %.Pearson’s chi-square or Fisher’s exact test was used to examine **categorical data**, and 2-sample t tests for continuous data.

* p< 0.05 for comparison between patients in the cirrhosis and control groups. † p< 0.05 for comparison between patients in the chronic hepatitis and control groups.
